# Modest Attenuation of HIV-1 Vpu Alleles Derived from Elite Controller Plasma

**DOI:** 10.1371/journal.pone.0120434

**Published:** 2015-03-20

**Authors:** Jingyan Chen, Nadine Tibroni, Daniel Sauter, Johanna Galaski, Toshiyuki Miura, Galit Alter, Birthe Mueller, Claudia Haller, Bruce D. Walker, Frank Kirchhoff, Zabrina L. Brumme, Takamasa Ueno, Oliver T. Fackler

**Affiliations:** 1 Department of Infectious Diseases, Integrative Virology, University Hospital Heidelberg, INF 324, Heidelberg, Germany; 2 German Center for Infection Research, Heidelberg University, Heidelberg. Germany; 3 Institute of Molecular Virology, Ulm University Medical Center, Ulm, Germany; 4 Institute of Tropical Medicine, Nagasaki University, Nagasaki, Japan; 5 Ragon Institute of Massachusetts General Hospital, Massachusetts Institute of Technology and Harvard University, Boston, Massachusetts, United States of America; 6 Division of Infectious Diseases, Massachusetts General Hospital, Boston, Massachusetts, United States of America; 7 Howard Hughes Medical Institute, Chevy Chase, Maryland, United States of America; 8 Faculty of Health Sciences, Simon Fraser University, Burnaby, British Columbia, Canada; 9 British Columbia Centre for Excellence in HIV/AIDS, Vancouver, British Columbia, Canada; 10 Center for AIDS Research, Kumamoto University, 2–2–1 Honjo, Kumamoto, Japan; 11 International Research Center for Medical Sciences (IRCMS), Kumamoto University, Japan; University of Pittsburgh Center for Vaccine Research, UNITED STATES

## Abstract

In the absence of antiretroviral therapy, infection with human immunodeficiency virus type 1 (HIV-1) can typically not be controlled by the infected host and results in the development of acquired immunodeficiency. In rare cases, however, patients spontaneously control HIV-1 replication. Mechanisms by which such elite controllers (ECs) achieve control of HIV-1 replication include particularly efficient immune responses as well as reduced fitness of the specific virus strains. To address whether polymorphisms in the accessory HIV-1 protein Vpu are associated with EC status we functionally analyzed a panel of plasma-derived *vpu* alleles from 15 EC and 16 chronic progressor (CP) patients. Antagonism of the HIV particle release restriction by the intrinsic immunity factor CD317/tetherin was well conserved among EC and CP Vpu alleles, underscoring the selective advantage of this Vpu function in HIV-1 infected individuals. In contrast, interference with CD317/tetherin induced NF-κB activation was little conserved in both groups. EC Vpus more frequently displayed reduced ability to downregulate cell surface levels of CD4 and MHC class I (MHC-I) molecules as well as of the NK cell ligand NTB-A. Polymorphisms potentially associated with high affinity interactions of the inhibitory killer immunoglobulin-like receptor (KIR) KIR2DL2 were significantly enriched among EC Vpus but did not account for these functional differences. Together these results suggest that in a subgroup of EC patients, some Vpu functions are modestly reduced, possibly as a result of host selection.

## Introduction

The clinical course and outcome of untreated HIV infection varies remarkably between patients. Typically, initially high viremia rapidly declines to a patient-specific setpoint level, followed by an extended clinically asymptomatic phase in which CD4+ T cell counts progressively decrease until acquisition of opportunistic infections marks the beginning of the symptomatic phase with high viral load. In rare cases, however, infected individuals remain asymptomatic with plasma virus loads below the limit of detection of conventional assays. Such patients are referred to as elite controllers (EC) [1,2]. The mechanisms controlling HIV infection in EC are multifactorial and the genetic disposition of the infected individual emerges as a defining parameter. Potent CD8+ cytotoxic T lymphocyte (CTL) responses as well as protective MHC-I alleles such as HLA-B*57 and B*27 are associated with elite control [3–6]. Additional mechanisms proposed for elite control include enhanced antibody-dependent cell mediated cytotoxicity (ADCC), antibody neutralization, and NK cell activity [7–10].

In addition to these host determinants, the course of infection is also determined by the fitness of the virus and indeed HIV-1 *gag*, *pol*, *env* and *nef* alleles isolated from EC patients display reduced biological activity in vitro [11–15] and *nef* genes are entirely disrupted in a subset of ECs [16,17]. Such reduction in function often reflects the acquisition of CTL escape mutations, indicating that the selection pressure on CTL evasion dominates that of viral protein function.

While the biological properties of Gag, Pol, Env and Nef proteins encoded by HIV-1 variants predominating in EC patients have been characterized [12–14], analogous information for the HIV-1 accessory protein Vpu is not available. Vpu is a 16kDa multifunctional protein encoded by HIV-1 and related primate lentiviruses. Initial studies of Vpu function revealed that the viral protein reduces the density of the HIV-1 entry receptor CD4 on the surface of infected cells by targeting it for degradation [18]. More recently, Vpu was identified as a potent antagonist of the host cell restriction factor CD317/tetherin which prevents release of infectious virions by tethering virus particles to the surface of virus producing cells [19,20]. Vpu is thought to counteract this inhibition by affecting the anterograde transport of the restriction factor [21] and its lateral displacement from viral budding sites [22]. CD317/tetherin also elicits proinflammatory signalling upon virion binding by triggering activation of the transcription factor NF-kB. Notably, this effect is also antagonized by Vpu [23]. Vpu downmodulation of cell surface levels of MHC-I and the NK cell activating ligand NTB-A has also been reported [24–26] and might contribute to evasion of HIV-1 infected cells from CTL and NK cell recognition. In addition, Vpu contains several HLA-A,-B, and -C restricted epitopes [27,28] and a polymorphism at residues 71 and 74 has been associated with high affinity interactions of the inhibitory killer immunoglobulin-like receptor (KIR) KIR2DL2 [9]. These various Vpu activities can be observed *in vitro* in HIV infected cells and are largely conserved among *vpu* alleles derived from longitudinal samples of HIV-1 infected patients with different courses of disease [29]. Considering the proposed roles of Vpu in governing HIV’s interaction with the host immune system we hypothesized that Vpu sequence and/or function may differ between HIV-1 variants predominating in EC and patients with classical disease progression (chronic progressors, CP). While Pickering and colleagues analyzed some Vpu functions in five long-tern non-progressor patients [29], not all of these individuals would be categorized as ECs based on their viral load and CD4 count and NK cell-related activities of Vpu were not addressed. We therefore revisited this issue and functionally characterized Vpu proteins from 15 EC and 16 CP patients, respectively.

## Materials and Methods

### Cells, plasmids, and reagents

A3.01, TZM-bl or HEK293T cells were cultivated in RPMI 1640 and DMEM, respectively, supplemented with 10% fetal calf serum, 1% penicillin-streptomycin (Invitrogen). The following reagent was obtained from the NIH AIDS Reagent Program, Division of AIDS, NIAID, NIH: A3.01 from Dr. Thomas Folks [30] and TZM-bl from Dr. John C. Kappes, Dr. Xiaoyun Wu and Tranzyme Inc. [31]. The proviral plasmids pHIV-1 NL4–3 wild type and pHIV-1 NL4–3Δ*vpu* have been described [32] and were originally provided by Valerie Bosch (DKFZ, Heidelberg).

Human CD317/Tetherin was cloned into the CMV promoter-based pCG expression vector coexpressing DsRed2. The NF-κB firefly luciferase reporter plasmid containing three NF-κB binding sites (pNF-κB(3x)-Firefly Luciferase) and an expression vector expressing a constitutively active mutant of IKKβ were kindly provided by Bernd Baumann. pTAL-Gaussia Luciferase, a minimal promoter reporter construct was purchased from Clontech (#631909) and used for normalization. It expresses gaussia luciferase under the control of the TATA-like promoter (pTAL) region from the Herpes simplex virus thymidine kinase (HSV-TK) that is not responsive to NF-κB.

The following antibodies were used: monoclonal mouse anti-transferrin receptor (Tfr) (clone H68.4, Zymed Laboratories); allophycocyanin (APC)-conjugated mouse-anti human CD4 antibody (clone RPA-T4; BD Bioscience); mouse anti-BST2/CD317 (clone 26F8; BD Bioscience); rabbit polyclonal anti-Vpu (Vpu-101AP; Fab Gennix); rat anti-GFP (clone 3H9; Chromotek); sheep anti-HIV-1 p24CA antiserum (from Barbara Müller, Department of Infectious Diseases, Heidelberg University Hospital); horseradish peroxidase-conjugated secondary antibodies (Dianova); SuperSignal West Pico Substrate (Thermo scientific).

### Study subjects

The EC and CP cohorts as well as were described in detail elsewhere [2,33]. Fifteen EC (median [interquartile range, IQR] CD4 counts 843 [654–955] cells/mm^3^, plasma viral load (pVL) <50 copies/ml) and sixteen CP (median [IQR] CD4 counts 284 [36.75–433] cells/mm^3^ pVL 107100 [62810–308200] copies/ml) were recruited in the present study. All EC and CP patients were infected with HIV-1 subtype B variants, treatment naive at the time of sample collection, recruited from the Boston area, and comparable with respect to ethnicity and date of HIV diagnosis (1985–2006 for EC vs. 1981–2003 for CP). All participants were recruited on the basis of viral load from outpatient clinics at local Boston hospitals and also referred from providers throughout the United States, after institutional review board approval and written informed consent. This study was approved by the Research Ethics Board at the Massachusetts General Hospital (Boston, MA) and all participants provided written informed consent.

### Viral RNA Isolation, RT-PCR Amplification and Vpu expression constructs

Procedures for isolation of viral RNA and RT-PCR amplification as well as the bulk sequence of plasma viral RNA were described previously [2]. Briefly, HIV-1 plasma virus from EC and CP patients was isolated by ultracentrifugation. Viral RNA was extracted by using the Qiagen viral RNA mini kit including on-column DNase treatment, eluted in DNase- and RNase-free water and stored at -80°C. HIV-1 gene regions were amplified using nested reverse transcriptase PCR (RT-PCR). No proviral amplification was observed in control reactions omitting the reverse transcription step. A minimum of three *vpu* clones were sequenced per patient and a single clone with an intact *vpu* reading frame that closely resembled the original bulk plasma RNA sequence was selected. Genbank accession numbers for these primary EC Vpu sequences are as follows: EC1: EU517748; EC2: EU517752; EC3: EU517730; EC4: EU517732; EC5: EU517737; EC6: EU517743; EC7: EU517745; EC8: EU517760; EC9: EU517759; EC10: EU517754; EC11: EU517757; EC12: EU517758; EC13: EU517722; EC14: EU517723; EC15: EU517724. CP Vpu sequences determined in this work were submitted to Genbank under the accession numbers from KM656058 to KM656073 (CP1–CP16). Vpu sequences were analyzed for recent ancestry by maximum-likelihood phylogenetic analysis as described [34] and *vpu* open reading frames were cloned into pEGFP and pIRES-EGFP expression vectors (Clontech).

### Expression of Vpu and Western blotting

TZM-bl cells were transfected with 3 μg Vpu expression plasmid using polyethylenimine (PEI), harvested 24 h post-transfection and lysed in SDS-sample buffer. Proteins were separated on 12.5% SDS-PAGE and blotted onto nitrocellulose membranes. Blocked membranes were probed with the following primary antibodies: rabbit polyclonal anti-Vpu (Vpu-101AP, Fab Gennix), rat anti-GFP (clone 3H9, Chromotek), monoclonal mouse anti-transferrin receptor (Tfr) (clone H68.4, Zymed Laboratories). Secondary antibodies were conjugated to horseradish peroxidase for ECL-based detection.

### Fluorescence microscopy

A3.01 T cells adhered to poly-lysine coated coverslips were fixed with 3% PFA. Coverslips were mounted in mowiol and analyzed with a Zeiss LSM510 confocal microscope with a 100x PLAN-APO objective lens. Images were recorded with the Zeiss proprietary software LSM5 and processed with Adobe Photoshop 4.0.

### Analysis of CD4 and CD317/tetherin modulation

To quantify CD4 and CD317 cell surface expression, TZM-bl cells were transfected with 3 μg expression construct for Vpu.GFP, VpuIRESGFP or GFP using polyethylenimine (PEI). 48 h post-transfection cells were washed and stained with allophycocyanin (APC)-conjugated mouse-anti human CD4 (clone RPA-T4; BD Bioscience) or with mouse anti-HM1.24/CD317/tetherin (clone 26F8; BD Bioscience) followed by staining with goat anti-mouse APC (115–136–071; Jackson ImmunoResearch). A FACS Calibur with BD CellQuest Pro 4.0.2 Software (BD Bioscience) and Cyflogic software were used for analysis. The MFI for surface-exposed receptors was quantified, relative CD4 and CD317/tetherin cell-surface expression levels were determined by calculating the ratio of the MFIs of high to low GFP expressing cell populations, and expressed relative to that of NL4.3 Vpu that was arbitrarily set to 100%.

### Analysis of MHC-I and NTB-A modulation

A3.01 T cells were electroporated with 30 μg expression plasmid and 48 h post-transfection the cells were washed and stained in PBS with allophycocyanin (APC)-conjugated mouse-anti human HLA-ABC antibodies (clone G46–2.6; BD Bioscience) or with mouse-anti NTB-A antibody (MAB19081; R&D) followed by staining with goat anti-mouse APC (115–136–071; Jackson ImmunoResearch). Relative MHC-I and NTB-A cell-surface expression levels were determined by calculating the ratio of the MFIs of high to low GFP expressing cell populations, and expressed relative to that of NL4.3 Vpu that was arbitrarily set to 100%.

### Virion infectivity assays

TZM-bl cells were seeded in 12-well plates (1x10^5^/well), and were co-transfected with 1.2 μg HIV-1NL4.3ΔVpu proviral DNA and 1.5 μg VpuIRESGFP expression construct or with empty vector pIRESGFP. TZM-bl cells co-transfected with 1.2 μg HIV-1NL4.3 wild type proviral DNA and 1.5 μg pIRESGFP served as positive control [32,35]. Two days post-transfection, 50 μl of the culture supernatants were added to TZM-bl reporter cells cultured in 96-well and the infectivity of HIV-1 was determined by analysis of firefly luciferase activity 72 h post infection.

### Antagonism of CD317-induced NF-ƙB signaling

293T cells were co-transfected with the following plasmids mediated by calcium phosphate in triplicate: 100 ng pNF-κB(3x)-Firefly Luciferase as NF-κB reporter; 25 ng pTAL (minimal promoter)-Gaussia Luciferase for normalization; 40 ng pCG-human tetherin IRES DsRed2; 100 ng indicated VpuIRESGFP expression vectors [36]. HIV-1 M WITO Vpu served as positive control in each experiment. Dual luciferase assays were performed 40 h post transfection and firefly luciferase signals were normalized to the corresponding gaussia luciferase signals.

### Statistical evaluation

All statistical analyses were conducted using GraphPad Prism 5 and statistical significance was determined using the Mann-Whitney U test (* p ≤ 0.05, ** p ≤ 0.01) or Fisher’s exact test as appropriate. Correlations between data sets were evaluated by applying Spearman’s correlation or Pearson’s correlation (* p ≤ 0.01, ** p ≤ 0.001; *** p ≤ 0.0001).

## Results

### Sequences and Expression Levels of Patient-derived Vpu Proteins

**Fig 1:**
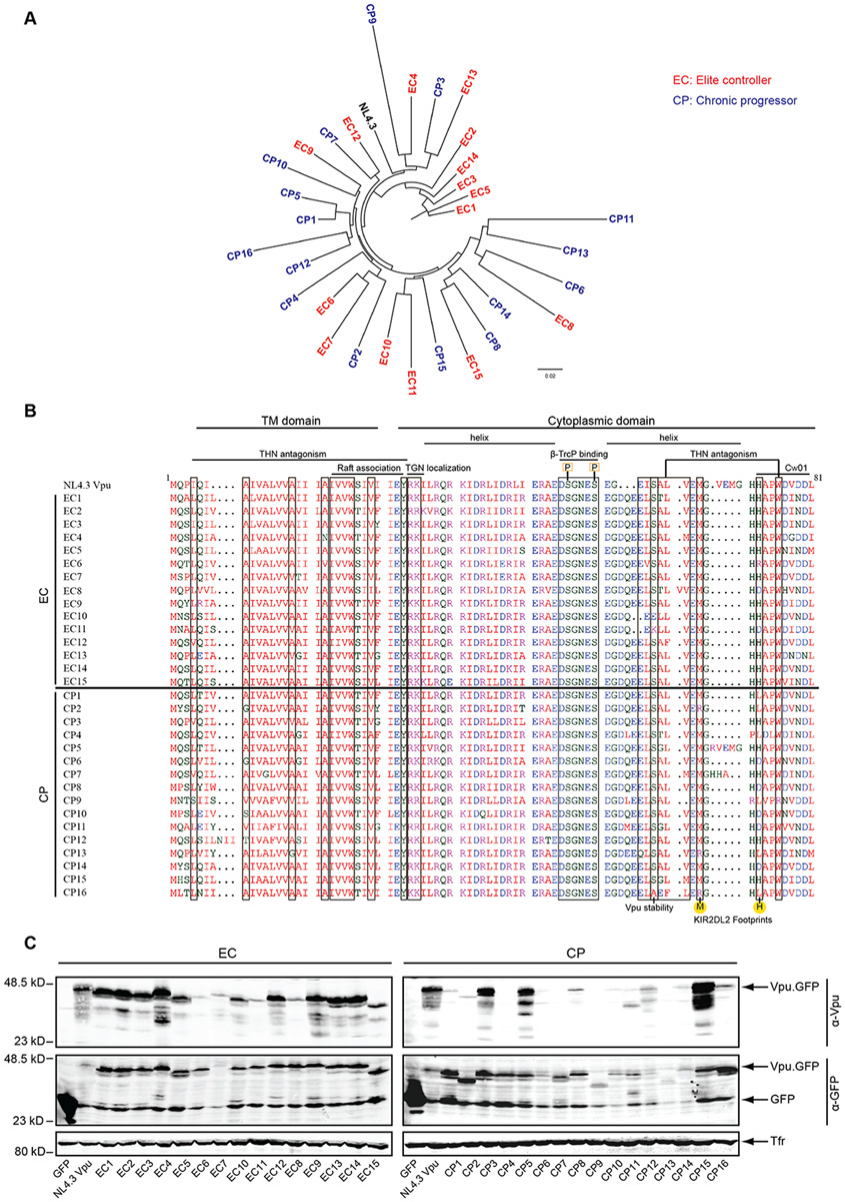
Maximum-likelihood phylogenetic tree, sequences alignment, expression and detection of plasma HIV RNA-derived Vpu clonal alleles. **A**) Maximum-likelihood phylogenetic tree of HIV-1 Vpu alleles. EC-derived Vpus in red, CP-derived Vpus in blue, and control strain NL4.3 in black. **B**) Amino acid sequence alignment of the Vpu proteins analyzed generated using Clustal Omega (EMBL-EBI). HIV-1 NL4.3 Vpu on the top serves as reference sequence. Boxes indicate the position of functionally relevant residues and motifs. Different colors present the properties of the amino acid: red for small and aromatic, blue for acidic, green for residues with hydroxyl or sulfhydryl groups and magenta for positively charged residues. *C*: Expression and detection of Vpu alleles. Lysates of A3.01 cells transfected with Vpu.GFP expression plasmids were analyzed by Western blotting using antibodies against Vpu, GFP, and transferrin receptor (Tfr).

For 15 EC and 16 CP patients, a plasma HIV RNA-derived *vpu* sequence with an intact open reading frame (ORF) representative of the patient’s bulk sequence was subcloned into pEGFP N1 and pIRESGFP vectors for functional analyses. Consistent with previous analyses of bulk plasma HIV RNA sequences from our EC cohort [2], clonal *vpu* sequences showed no gross defects or recent shared ancestry (Fig. 1A). Amino acid sequence variation among individual Vpu variants for both groups ranged from 16% to 24% deviation from group’s consensus sequence and sequence alignment did not reveal signature motifs specific for EC or CP Vpu variants (Fig. 1B). Amino acid motifs previously identified as determinants for aspects of Vpu’s biological activity such as interaction with CD317/tetherin [22,37], localization of lipid raft microdomains [38], TGN localization [39], interaction with the endocytic machinery of the host cell (ExxxLV motif) [40], or Vpu stability (S61) [41] were generally well conserved across all Vpu variants. However some Vpus contained changes in individual residues related to CD317/tetherin antagonism (EC4, EC6, EC7, EC11, EC12, CP3, CP7, CP9, CP13 and CP15) or lacked the S61 residue (CP16) (Fig. 1B). The DS_52_XXES_56_ motif which, in its phosphorylated state, recruits β-TrCP to target cargo such as CD4 and CD317/tetherin for proteasomal degradation, was fully conserved in all Vpu variants studied. In addition to functional motifs, Vpu also contains determinants for immune recognition such as the CTL recognition epitopes restricted by HLA-A*33:*03* (EYRKILRQR) and by HLA-Cw*01:*02* (HAPWDVNDL) (residues 28–34 and 73–81 in HIV-1 NL4–3 Vpu, respectively) [27,42]. While the EYRKILRQR epitope was very well conserved across all patient-derived Vpu proteins, mutations in the HLA-C restricted HAPWDVNDL epitope were more frequent in CP than in EC Vpus (e.g. amino acid position 73 was changed from the typical histidine to another residue in 11 out of 16 CP Vpus but only in 3 out of 15 EC Vpus, p = 0.011, Fisher’s exact test, two-tailed). This histidine together with a methionine three residues upstream has also been identified as a polymorphism that determines the affinity of interactions KIR2DL2 on NK cells with HIV-infected cells [9] (henceforth M/H polymorphism: positions 70 and 73 in NL4.3 Vpu and most patient-derived Vpu proteins analyzed here, positions 71 and 74 in [9]). While M/H genotypes are associated with high affinity binding and thus evasion from NK-cell mediated lysis, changes in this motif decrease binding of inhibitory KIRs. This M/H genotype was significantly more frequent in the EC (12/15) than in CP (5/16) Vpus analyzed herein (p = 0.011, Fisher’s exact test, two-tailed).

We next sought to evaluate the expression levels of our panel of patient-derived Vpu variants (Fig. 1C). When expressed as Vpu.GFP fusion, full length protein was detected with an anti-GFP antibody for most Vpus except for EC6, CP13 and CP14, for which only weak signals could be detected by Western blotting. Detection with a Vpu specific antibody revealed robust expression of most EC Vpus but detection of CP Vpus was substantially less frequent. Consistently, analyses of non-fusion protein encoded by IRESGFP plasmids (S1 Fig) revealed expression for most EC but not CP Vpus included in this study, despite comparable amounts of GFP expression in all samples. The anti-Vpu antibody used was raised against a peptide from amino acids 58–80 of NL4–3 Vpu and the lack of detection of individual Vpu variants correlated well with sequence variation in this region. Finally, all Vpu.GFP proteins analyzed displayed similar subcellular localization with a strong enrichment at an intracellular membrane compartment and a subpopulation within dotted structures at the plasma membrane (S2 Fig).

### CP Vpus Downregulate Cell Surface CD4 More Potently Than EC Vpus

**Fig 2:**
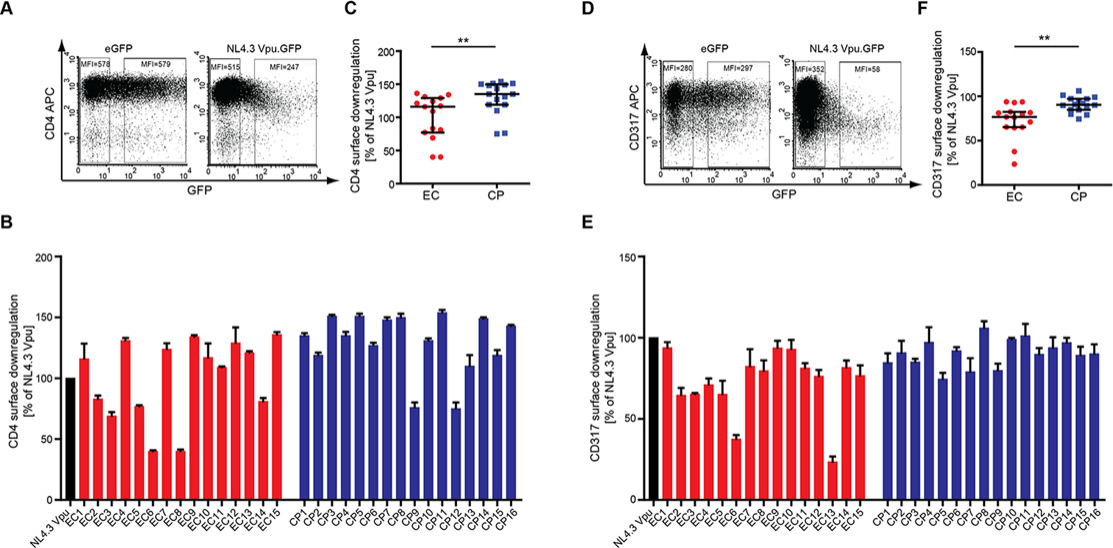
CD4 and CD317/tetherin downregulation by patient-derived Vpus. Surface CD4 and CD317/tetherin levels were analyzed by flow cytometry on A3.01cells 48 h post transfection with the indicated Vpu.GFP expression constructs. **A**) Representative flow cytometry dot plots of gated living cells for CD4-APC (y-axis) vs. GFP (x-axis). The MFI (Y Geo Mean) of untransfected (left gate) and medium to high GFP-expressing (right gate) cells is indicated. CD4 cell surface levels of transfected cells were normalized to untransfected cells in the same sample by calculating the ratio of the MFIs of left and right gates and expressed relative to that of NL4.3 Vpu that was arbitrarily set to 100%. **B**) CD4 cell surface levels of A3.01cells expressing patient-derived Vpu proteins relative to NL4.3 Vpu. Shown are mean values of triplicate transfections with the indicated standard deviation. The result is representative of three independent experiments. **C**) Comparison of CD4 cell surface levels in A3.01 cells expressing EC or CP Vpu alleles. Statistical significance was assessed using two-tailed Mann-Whitney U-Test (p = 0.02), bars represent median and interquartile ranges. **D**) Representative flow cytometry dot plots of gated living cells for CD317/tetherin-APC (y-axis) vs. GFP (x-axis). The MFI (Y Geo Mean) of untransfected (left gate) and medium to high GFP-expressing (right gate) cells is indicated. CD317/tetherin cell surface levels of transfected cells were normalized to untransfected cells in the same sample by calculating the ratio of the MFIs of left and right gates and expressed relative to that of NL4.3 Vpu that was arbitrarily set to 100%. **E**) CD317/tetherin cell surface levels of TZM-bl cells expressing patient derived Vpu proteins relative to NL4.3 Vpu. Shown are mean values of triplicate transfections with the indicated standard deviation. The result is representative of three independent experiments. **F**) Comparison of CD317 cell surface levels in A3.01 cells expressing EC or CP Vpu alleles. Statistical significance was assessed using two-tailed Mann-Whitney U-Test (p = 0.64), bars represent median and interquartile ranges.

To characterize the biological properties of EC and CP Vpus, these proteins were transiently expressed and subjected to quantitative analysis of established Vpu activities. We first assessed the ability of Vpu to reduce cell surface levels of CD4 in A3.01 cells upon transient transfection of the respective Vpu.GFP expression plasmids. GFP expression allowed to identify transfected cells and to calculate the relative CD4 downregulation activity as the ratio in CD4 mean fluorescence intensity (MFI) between GFP negative and positive cells. The extent of cell surface CD4 downregulation by NL4.3 Vpu (Fig. 2A) was arbitrarily set to 100% and the values obtained upon expression of Vpu alleles expressed relative to this. All patient-derived Vpu.GFP variants displayed robust CD4 downregulation activity (Fig. 2B), albeit with variable efficiency. While almost all CP Vpus analyzed were similarly active in CD4 downmodulation as NL4.3 Vpu (14 of 16), Vpus with intermediate CD4 downregulation activity relative to NL4.3Vpu were more frequent in the EC Vpu group (6 of 15). This resulted in a statistically significant difference between CD4 downregulation by EC (median CD4 downregulation activity relative to NL4.3 116.0% [Interquartile Range (IQR) 77.0%-129.0%]) and CP Vpus (median CD4 downregulation activity relative to NL4.3 135.0% [IQR 119.0%-149.8%]) (p< 0.009; Fig. 2B, 2C). Vpus with slightly reduced CD4 downregulation activity are thus more frequent among ECs than CPs when expressed in A3.01 cells.

### Downregulation of Cell Surface CD317/tetherin, MHC-I and NTB-A by EC and CP Vpus

We next conducted analyses analogous to those for CD4 for three additional cell surface receptors: CD317/tetherin, MHC-I and NTB-A. Similar to the results for CD4, CD317/tetherin cell surface exposure was markedly reduced by NL4.3Vpu.GFP and this downregulation was well conserved among all CP Vpu variants with individual alleles displaying slightly higher or lower activity, respectively, than NL4.3 Vpu (Figs. 2D, 2E) (median CD317/tetherin downregulation activity relative to NL4.3 90.6% [IQR 84.8%-97.1%]). CD317/tetherin downregulation by EC Vpus was overall less efficient and 2 of 15 EC Vpus were markedly impaired in this activity (Fig. 2F) (median CD317/tetherin downregulation activity relative to NL4.3 76.6% [IQR 65.2%-82.4%]; EC vs. CP Vpus *p = 0*.*002*, two-tailed Mann-Whitney U-Test).

**Fig 3:**
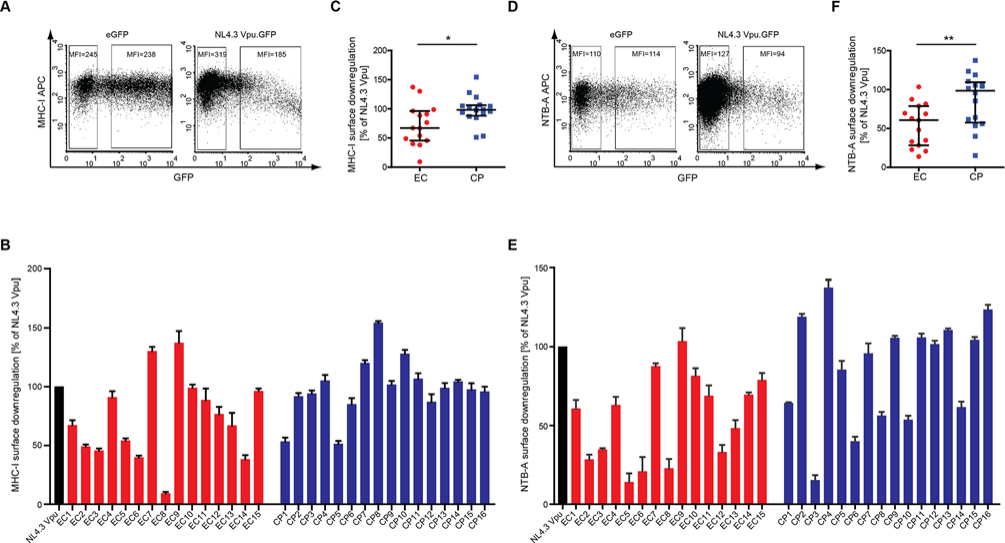
MHC-I and NTB-A downregulation by patient-derived Vpus. Surface MHC-I and NTB-A levels were analyzed by flow cytometry on A3.01 cells 48 h post transfection with the indicated Vpu.GFP expression constructs. **A**) Representative flow cytometry dot plots of gated living cells for MHC-I-APC (y-axis) vs. GFP (x-axis). The MFI (Y Geo Mean) of untransfected (left gate) and medium to high GFP-expressing (right gate) cells is indicated. MHC-I cell surface levels of transfected cells were normalized to untransfected cells in the same sample by calculating the ratio of the MFIs of right and left gates and expressed relative to that of NL4.3 Vpu that was arbitrarily set to 100%. **B**) MHC-I cell surface levels of A3.01 cells expressing patient derived Vpu proteins relative to NL4.3 Vpu. Shown are mean values of triplicate transfections with the indicated standard deviation. The result is representative of three independent experiments. **C**) Comparison of MHC-I cell surface levels in A3.01 cells expressing EC or CP Vpu alleles. Statistical significance was assessed using two-tailed Mann-Whitney U-Test (p = 0.02), bars represent median and interquartile ranges. **D**) Representative flow cytometry dot plots of gated living cells for NTB-A-APC (y-axis) vs. GFP (x-axis). The MFI (Y Geo Mean) of untransfected (left gate) and medium to high GFP-expressing (right gate) cells is indicated. NTB-A cell surface levels of transfected cells were normalized to untransfected cells in the same sample by calculating the ratio of the MFIs of right and left gates and expressed relative to that of NL4.3 Vpu that was arbitrarily set to 100%. **E**) NTB-A cell surface levels of A3.01 cells expressing patient derived Vpu proteins relative to NL4.3 Vpu. Shown are mean values of triplicate transfections with the indicated standard deviation. The result is representative of three independent experiments. **F**) Comparison of NTB-A cell surface levels in A301 cells expressing EC or CP Vpu alleles. Statistical significance was assessed using two-tailed Mann-Whitney U-Test (p = 0.01), bars represent median and interquartile ranges.

Similar trends for the activity of EC and CP Vpus were also observed when their ability to reduce cell surface MHC-I and NTB-A levels was analyzed. NL4.3 Vpu.GFP displayed a moderate yet significant reduction of cell surface MHC-I (Fig. 3A-C) and NTB-A (Fig. 3D-F). Patient-derived Vpu alleles also caused a moderate reduction of MHC-I cell surface exposure that was often even less pronounced than that of NL4–3 Vpu (Fig. 3B). Such reduced MHC-I cell surface downregulation was more frequent among EC (6 of 15) than CP (2 of 16) Vpus, resulting in a statistically significant difference between both groups (Fig. 3C, p = 0.02). Comparable results were obtained using VpuIRESGFP expression constructs (data not shown). Similarly, cell surface downmodulation of NTB-A was observed for many of the patient-derived Vpu variants, however loss of NTB-A downregulation was more frequent in the EC Vpu group (9 of 16 vs. 6 of 16), which displayed statistically significant lower NTB-A downregulation activity than CP Vpus (Fig. 3D-F, p = 0.01).

### Downregulation of Cell Surface CD4 and CD317/tetherin by Vpu expressed from IRESGFP constructs

In addition to the analysis of Vpu.GFP fusion proteins in A3.01 cells we also analyzed downregulation of cell surface CD4 and CD317/tetherin for Vpu proteins expressed from VpuIRESGFP constructs in TZM-bl cells (S3 Fig). Expression of VpuIRESGFP resulted in fewer GFP positive cells with however more pronounced cell surface receptor downregulation than observed with GFP fusion proteins. These differences likely reflect (i) the increased rate of CMV-driven expression of Vpu over IRES-driven expression of GFP in the IRES-GFP context, which may yield higher Vpu concentrations per GFP positive cell than upon expression of Vpu.GFP proteins and/or (ii) differences between the A3.01 and TZM-bl cells used. In the case of CD4 downregulation, similar differences between EC and CP Vpu in CD4 downregulation as observed with Vpu.GFP fusion proteins were detected with VpuIRESGFP expression plasmids (S3A-C Fig, *p*<0.05). In contrast, the more efficient downregulation of CD317/tetherin observed with Vpus expressed from IRES-GFP plasmids resulted in less pronounced differences between individual alleles. Even though reduced downregulation activity was observed for the same alleles (EC6 and EC13), differences between the EC and CP groups were not statistically significant (*p* = 0.64, two-tailed Mann-Whitney U-Test). We speculate that per cell concentrations of Vpu are more limiting for the mechanisms employed to downregulate cell surface CD317/tetherin than CD4. In this scenario, high levels of Vpu expression may compensate or limit subtle functional deficits of individual Vpu variants in CD317/tetherin downregulation. Irrespective of the Vpu expression system used, the physiological relevance of differences in the biological activity of Vpu alleles observed will need to be validated in future infection experiments of primary human target cells.

### CD317 Antagonism is conserved between EC and CP Vpus

**Fig 4:**
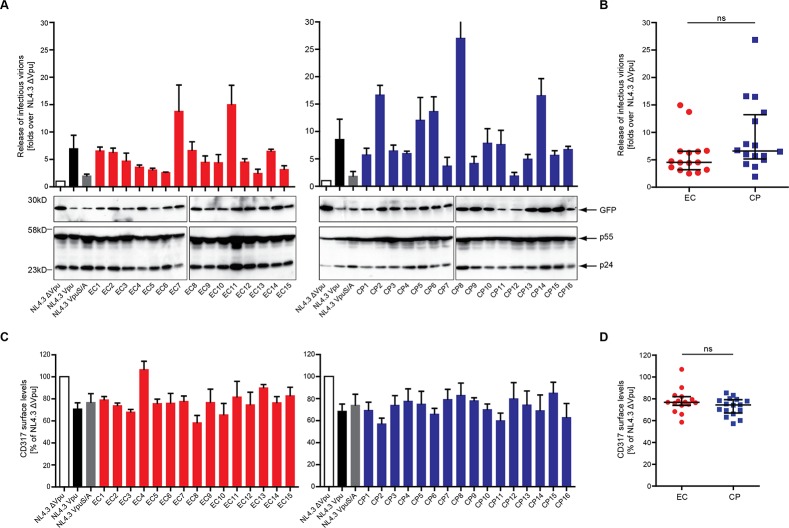
Recovery of HIV-1 particle release by patient-derived Vpus. TZM-bl cells were transiently transfected with HIV-1NL4.3 Δvpu provirus plasmids and the indicated Vpu allele or controls. 48 h post transfection, the resulting viral supernatants were assayed for infectivity on TZM-bl indicator cells to determine the amount of infectious virions produced. TZM-bl cell lysates from one replicate of the assay were subjected to Western blot detection, and TZM-bl cells from the same replicate were harvested and assayed for cell-surface level of CD317/tetherin. **A**) The yield of infectious HIV-1 in the supernatant and cell-associated levels of GFP, p24CA, and p55Gag were analyzed. HIV-1 particle release in the supernatant was assessed by measuring the induction of Luciferase units (top) in infected TZM-bl cells. Values (y axis) are normalized to that of control NL4.3Δ*vpu*, which was set to 1, error bars represent the standard error of the mean (SEM) for three independent experiments. Western blot results show the expression level of GFP, p24 and p55 in one representative experiment. **B**) Comparison of enhancement of virion release mediated by EC and CP Vpu alleles. Statistical significance was assessed using two-tailed Mann-Whitney U-Test (p = 0.07), bars represent median and interquartile ranges. **C**) CD317/tetherin cell surface downregulation by patient Vpu. TZM-bl cells transfected with HIV-1 NL4.3 Δvpu and the indicated VpuIRESGFP plasmids were harvested 48 h post transfection and CD317/tetherin cell surface levels quantified by flow cytometry. The y-axis represents the relative CD317/tetherin cell surface levels remaining normalized to control cells transfected with NL4.3Δ*vpu* and IRESGFP (set to 100%). **D**) Comparison of CD317/tetherin cell surface levels in TZM-bl cells producing viral particles and expressing EC or CP Vpu alleles. Statistical significance was assessed using two-tailed Mann-Whitney U-Test (p = 0.29), bars represent median and interquartile ranges.

We next examined if patient-derived Vpu alleles maintain the ability to overcome the particle release restriction imposed by CD317/tetherin and thus to rescue HIV-1 particle release. CD317-positive TZM-bl cells were co-transfected with *vpu*-deficient proviral DNA (HIV-1 NL4.3 Δ*vpu*) and VpuIRESGFP expression plasmids and the amount of infectious HIV released in the cell culture supernatant was assessed as correlate for HIV particle release (Fig. 4A and B). Virus producing cells were harvested and analyzed for levels of cell-associated Gag (Fig. 4A, lower panel) and CD317/tetherin cell surface density (Fig. 4C, D). Although to a lower extent than observed for isolated Vpu expression (S3 Fig), cell surface CD317/tetherin levels were reduced in presence of NL4.3 Vpu as well as by the mutant NL4.3 VpuS/A, in which the di-serine motif required for interactions with ß-TrCP is disrupted [43]. Reflecting efficient CD317/tetherin antagonism, NL4.3 Vpu but not its di-serine mutant significantly increased the production of infectious progeny of HIV-1 Δ*vpu*. Almost all patient-derived Vpu alleles displayed CD317/tetherin antagonizing activity above that of NL4.3 VpuS/A, however to greatly varying extents. While the activity of most alleles was comparable to that of NL4.3 Vpu, several naturally occurring variants promoted a more substantial production of infectious HIV-1 than the prototypic variant NL4.3. A trend towards a higher frequency of Vpu alleles with at least two-fold higher activity than NL4.3Vpu was observed in the CP cohort, although the difference between both groups was not statistically significant (4.6 [IQR 3.2–6.6] and 6.7 [IQR 5.2–13.3] fold over HIV-1 NL4.3 Δ*vpu* for EC and CP Vpus, p = 0.07) (Fig. 4B). Similar to the previous results with isolated Vpu expression (S3 Fig), reduction of CD317/tetherin surface levels on virus producing cells was well conserved among the Vpu variants analyzed and did not differ between EC and CP Vpus (Fig. 4C). No direct overall correlation was observed between downmodulation of CD317/tetherin cell surface and enhancement of virion release (Pearson’s correlation R = 0.35; p = 0.05, data not shown). Furthermore, CD317/tetherin downmodulation by alleles with pronounced activity in particle release (e.g. CP8, EC11) was comparable to that of all other Vpus analyzed and the CD317/tetherin downregulation deficient variant EC4 enhanced particle release to similar extent to many other alleles with normal CD317/tetherin downregulation activity. Antagonizing CD317/tetherin to enhance HIV-1 particle release thus represents a conserved activity of EC and CP Vpus which, however, does not correlate with the extent of removal from the cell surface of the restriction factor.

### Reduction of NF-κB Activation Induced by CD317/tetherin is Poorly Conserved Among EC and CP Vpus

**Fig 5:**
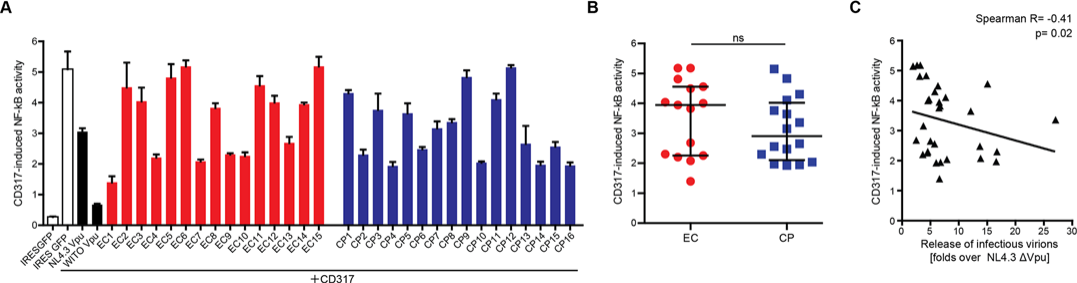
Antagonism of CD317/tetherin-induced NF-kB signaling. **A**) CD317/tetherin-induced NF-κB activation by Vpu alleles. Luciferase activity of the NF-κB reporter was determined 40 h post transfection of 293T cells with expression plasmids for CD317/tetherin or filler, the indicated VpuIRESGFP expression plasmids and luciferase reporter plasmids. HIV-1 NL4.3 Vpu and HIV-1 M WITO Vpu served as controls. Mean values of 6–9 transfections are shown with the indicated standard deviation. **B**) Effect of EC and CP Vpu alleles on CD317/tetherin-induced NF-κB activation. Statistical significance was assessed using two-tailed Mann-Whitney U-Test (p = 0.29), bars represent median and interquartile ranges. **C**) Correlation of Vpu-mediated inhibition of CD317/tetherin-induced NF-κB activity and release of infectious virions. Statistical analyses were done using Spearman’s correlation.

As final functional parameter we assessed the ability of patient-derived Vpus to interfere with activation of NF-κB triggered by CD317/tetherin (Fig. 5). Vpu inhibits canonical NF-κB signalling by directly targeting CD317/tetherin but also independently of this restriction factor [36,44]. When expressed in 293T cells together with an NF-κB luciferase reporter, CD317/tetherin triggered a robust activation of NF-κB (Fig. 5A, compare white bars). While NL4–3 Vpu only had moderate effects, WITO Vpu derived from a transmitted/founder subtype B HIV-1 group M strain potently suppressed CD317/tetherin-induced NF-κB activation as recently reported [36]. Such potent inhibition was not observed for any of the patient-derived Vpu alleles, many of which failed to inhibit NF-kB activation (Fig. 5A). This Vpu activity was similarly little conserved among EC and CP Vpus (Fig. 5A) (median 3.9 [IQR 2.2–4.6] and 2.9 [2.1–4.0] fold of remaining NF-kB firefly luciferase activation over Gaussia luciferase activity for EC and CP Vpus, respectively). Moreover, no strong correlation was observed between the ability of Vpu to interfere with CD317/tetherin-induced reduced NF-κB activation and to antagonize the particle release restriction by CD317 (Fig. 5C) (Spearman’s correlation R = -0.41, p = 0.02n) or to downmodulate CD317/tetherin cell surface levels (data not shown). Suppression of NF-κB activation was therefore not well conserved among the Vpus derived from EC and CP patient cohorts.

## Discussion

The aim of this study was to compare Vpu sequence and function between alleles derived from EC and CP HIV patient cohorts to gain insight into which Vpu activities may contribute to HIV pathogenesis (see summary of all results in Table 1). Within the limited sample size of 15 and 16 *vpu* alleles derived from EC and CP patients, respectively, our results did not reveal a significant difference between EC and CP Vpus in their ability to antagonize the CD317/tetherin-mediated restriction to HIV particle release. However, owing to a higher frequency of alleles with significantly reduced activity in this group, EC Vpus on average displayed reduced activity in downregulating CD4, MHC-I and NTB-A cell surface levels compared to CP Vpus. These functional differences suggest that at least in some EC HIV patients, Vpu function is slightly attenuated, possibly reflecting host selection.

**Table 1:** Summary of Vpu allele function, HLA background and HLA or KIR associated amino acid polymorphisms. The activity of patient derived *vpu* alleles was rated relative to that of NL4.3 Vpu and the following activity classes were defined: CD4 and CD317/tetherin: +: more than 80% of NL4.3 Vpu activity, +/-: 50% - 80% of NL4.3 Vpu activity, -: lower than 50% activity of NL4.3 Vpu. MHC-I and NTB-A downregulation: +: more than 75% activity of NL4.3 Vpu, +/-: 75% - 50% of NL4.3 Vpu activity, -: lower than 50% activity of NL4.3 Vpu. Virion release: ++: fold increase over NL4.3Δvpu > 12, +: fold increase over NL4.3Δvpu > 3, -: fold increase over NL4.3Δvpu < 3. Interference with NF-kB activation by CD317/tetherin: +: equally or more active than NL4.3 Vpu (significant difference to IRESGFP), p > 0.001, -: inactive (not different from IRESGFP, p> 0.01).

EC	CD4↓	CD317↓	MHC-1↓	NTB-A↓	Release assay	NF-κB CD317	NF-κB IKKβ	HLA C		KIR-associated polymorphism		KIR2DL2
								C1	C2	71M	74H	
**EC1**	**+**	**+**	**+/-**	**+/-**	**+**	**+**	**+**	C0401	C1701			-
**EC2**	**+**	**+**	-	-	**+**	-	-	C0202	C0701			u.d.
**EC3**	**+**	**+**	-	-	**+**	-	-	C0701	C0602			-
**EC4**	**+**	**+/-**	**+**	**+/-**	**+**	**+**	**+**	C0304	C1202			+
**EC5**	**+**	**+**	**+/-**	-	**+**	-	-	C0304	C0602			-
**EC6**	**+/-**	**+**	-	-	**+**	-	-	C0401	C1402	71M	74R	u.d.
**EC7**	**+**	**+**	**+**	**+**	**++**	**+**	**+**	C0202	C1203			-
**EC8**	**+/-**	**+**	-	-	**+**	-	-	C0205	C0303	71M	74D	u.d.
**EC9**	**+**	**+**	**+**	**+**	**+**	**+**	-	C04	C12			+
**EC10**	**+**	**+**	**+**	**+**	**+**	**+**	-	C1601	C1601			u.d.
**EC11**	**+/-**	**+**	**+**	**+/-**	**++**	-	-	C0702	C0401	71M	74D	+
**EC12**	**+**	**+**	**+**	-	**+**	-	-	C0102	C0602			u.d.
**EC13**	**+**	**+/-**	-	-	-	**+**	-	C1402	C1801			u.d.
**EC14**	**+**	**+**	**+/-**	**+/-**	**+**	-	-	C0401	C0701			u.d.
**EC15**	**+**	**+**	**+**	**+**	**+**	-	-	C0802	C1601			u.d.
**CP**												
**CP1**	**+**	**+**	**+/-**	**+/-**	**+**	-	**+**	C03	C08	71M	74L	+
**CP2**	**+**	**+**	**+**	**+**	**++**	**+**	-	C04	C07	71R	74L	u.d.
**CP3**	**+**	**+**	**+**	-	**+**	-	-	C05	C15			u.d.
**CP4**	**+**	**+**	**+**	**+**	**+**	**+**	**+**	C07	C07	71M	74L	u.d.
**CP5**	**+**	**+**	**+/-**	**+**	**+**	-	-	C03	C04			-
**CP6**	**+**	**+**	**+**	-	**+**	**+**	-	C0202	C06	71M	74D	u.d.
**CP7**	**+**	**+**	**+**	**+**	**+**	**+**	-	C04	C05			-
**CP8**	**+**	**+**	**+**	**+/-**	**++**	**+**	-	C01	C07	71M	74D	u.d.
**CP9**	**+**	**+/-**	**+**	**+**	**+**	-	-	C04	C07	71M	74L	+
**CP10**	**+**	**+**	**+**	**+/-**	**+**	**+**	-	C02	C12	71M	74D	-
**CP11**	**+**	**+**	**+**	**+**	**+**	-	-	C07	C08	71M	74D	-
**CP12**	**+**	**+**	**+**	**+**	-	-	-	C02	C04			-
**CP13**	**+**	**+**	**+**	**+**	**+**	**+**	-	C02	C04	71R	74L	u.d.
**CP14**	**+**	**+**	**+**	**+/-**	**++**	**+**	**+**	C04	C07	71M	74D	u.d.
**CP15**	**+**	**+**	**+**	**+**	**+**	**+**	**+**	C096	C07			u.d.
**CP16**	**+**	**+**	**+**	**+**	**+**	**+**	**+**	C07	C07	71R	74L	u.d.

The activity of patient derived *vpu* alleles was rated relative to that of NL4.3 Vpu and the following activity classes were defined: CD4 and CD317/tetherin: +: more than 80% of NL4.3 Vpu activity, +/-: 50%- 80% of NL4.3 Vpu activity, -: lower than 50% activity of NL4.3 Vpu. MHC-I and NTB-A downregulation: +: more than 75% activity of NL4.3 Vpu, +/-: 75%- 50% of NL4.3 Vpu activity, -: lower than 50% activity of NL4.3 Vpu. Virion release: ++: fold increase over NL4.3Δvpu ˃ 12, +: fold increase over NL4.3Δvpu ˃ 3, -: fold increase over NL4.3Δvpu < 3. Interference with NF-kB activation by CD317/tetherin: +: equally or more active than NL4.3 Vpu (significant difference to IRESGFP), p ˃ 0.001, -: inactive (not different from IRESGFP, p˃ 0.01).

The characterization of Vpu alleles isolated from EC and CP patient cohorts provided insights into the conservation of Vpu functions. Importantly, tetherin antagonism was well conserved in EC and CP Vpus, suggesting that the variations in Vpu expression levels detected by Western Blotting do not predict the activity of individual Vpu alleles. The strict conservation of CD317/tetherin antagonism among the Vpu alleles analyzed confirms that this Vpu activity is under strong selection and supports the concept that CD317/tetherin counteraction represents a cardinal *in vivo* function of Vpu [29,45–48]. The high degree of conservation of CD317/tetherin antagonism among EC Vpus also refutes the hypothesis that the EC status of the patients analyzed may be the consequence of a loss of tetherin antagonism. This strong selection pressure on CD317/tetherin antagonism likely mirrors the need to surmount the particle release restriction it imposes for efficient virus replication but may additionally reflect the recently described protection of infected cells from ADCC [49–51]. In contrast, interference with NF-κB activation by CD317/tetherin was poorly conserved in both Vpu groups, suggesting that this Vpu activity may not be of major relevance at the stages of chronic HIV infection analyzed here. While the conservation of antagonism of the particle release restriction is consistent with a recent study by Pickering and colleagues, interference with CD317/tetherin-mediated signalling was much more conserved in longitudinal samples of patients with different disease progression [29]. Considering that the Vpu alleles analyzed here were obtained from patients with established chronic or controlled infections, our results do not exclude that interference with innate immune signalling by Vpu represents an important function during the acute phase of infection that can subsequently be lost. Consistently, Pickering *et al*., observed in some patients a trend towards elevated interference with CD317/tetherin-mediated signalling for Vpus isolated early post seroconversion that was reduced at later stages of HIV infection [29]. In contrast, downregulation of cell surface CD4, MHC-I and NTB-A was overall less efficient in the EC Vpu group relative to CP Vpus, indicating that Vpu adaptation in some EC patients is paralleled with reduced biological activity, which could potentially contribute to increased host control of virus replication. However, this reduced activity on its own will most likely not have major impact in the context of infected cells as it can in principle be compensated by other viral gene products (Nef and Env for CD4 downregulation [52,53]; Nef for MHC-I downregulation [54], Nef and Vpr for evasion from NK cell killing [55–58]). Since Nef and Env function can also be compromised in EC patients [13,14], it will be of interest for future studies to compare sequence and function of all these genes within individual patients to dissect the overall fitness cost of the virus, ideally in the context of patient-derived infectious molecular clones.

In addition to the question of how adaptation of Vpu may be associated with elite control of HIV replication, this analysis allows us to draw conclusions on the interconnections between individual Vpu activities in CD317/tetherin antagonism and their molecular determinants. Consistent with increasing evidence in the literature, net levels of cell surface downregulation were not predictive of Vpu’s ability to counteract CD317/tetherin-mediated restrictions to particle release [35,59,60]. Similarly, the lack of correlation between CD317/tetherin antagonism and the ability to inhibit CD317/tetherin-induced NF-κB activation confirms that these two Vpu activities are mechanistically uncoupled [23,61,62]. With respect to the mechanism by which Vpu antagonizes the particle release restriction by CD317/tetherin, these results are consistent with the model that Vpu affects CD317/tetherin during its anterograde transport to the plasma membrane to alter its lateral distribution of the restriction factor in specific sub-membrane microdomains [21,59,22]. While the loss of this activity for some *vpu* alleles studied is explained by disruption of critical motifs (e.g. disruption of tetherin interaction by A19N in EC4 or disruption of the ExxxLV motif in CP9 [37,40,63], the reasons for the reduced CD317/tetherin antagonism of alleles such as EC13 or CP12 are less obvious. These alleles may prove valuable tools for further dissecting the molecular mechanism of CD317/tetherin antagonism.

An important question raised by our results relates to the nature of the selection pressure(s) that may drive Vpu adaptation in EC patients and their impact on Vpu function. The main difference between EC and CP Vpu sequences we observed was in the enrichment of the KIR2DL2 footprint M/H polymorphism in EC Vpus. It was therefore tempting to speculate that the functional differences observed reflect an adaptation of HIV-1 to NK cell-mediated immune pressure, which is expected to facilitate interaction with inhibitory KIR2DL2 and thus suppress antiviral NK responses [9]. However, within the limited number of patients for which KIR2DL2 genotyping was available, no strict correlation between presence of KIR2DL2 and occurrence of the M/H polymorphism was observed (Table 1), indicating that KIR2DL2-independent processes may also lead to selection of this genotype. Irrespective of the KIR2DL2 genotype, the presence of the M/H polymorphism did not explain the functional differences observed between EC and CP Vpus (Table 1 and data not shown). In this context it is important to note that due to the overlap of *vpu* and *env* open reading frames, concomitant to polymorphisms in Vpu, Env sequences are also changed. This results in alterations in the Env signal peptide that are enriched in EC (93%, 14/15) vs. CP (56%, 9/16) and it will be interesting to investigate whether this Env polymorphism is associated with functional differences.

As discussed above, immune pressure in addition to suppression of antiviral NK responses likely contribute to the enrichment of the M/H polymorphism in EC Vpus. Among the Vpus studied, the majority of differences concerned H73 while M70 was largely conserved in both Vpu groups. Since H73 is also part of an epitope for CTL recognition, polymorphisms at this position may reflect evasion from CTL recognition. Consistent with the observed enrichment of H73 in EC Vpus, CTL escape mutations are significantly more frequent in the CP than the EC patient cohorts studied [2]. On the other hand, such CTL escape mutations may affect binding to inhibitory KIRs and/or alter peptide processing and presentation, thereby rendering infected cells susceptible to NK cell lysis. In this scenario, the relative potency of CTL vs. NK cell responses in each infected individual would determine which viral sequence is selected. The ability of Vpu to tolerate various amino acids at this position without notable reduction in any of the activities analyzed here thus may provide the virus with the versatility to adjust to the environment in its immunocompetent host. Finally, Vpu contains epitopes mediating ADCC that remain to be precisely defined [64], suggesting that evasion from ADCC may represent an additional driving force for *vpu* sequence evolution. Of note, adaptation of *vpu* sequences (and that of other viral genes) to this elevated immune pressure in EC patients does not result in escape from elite control indicating that host determinants, possibly including efficient NK cells responses and elevated HLA-C expression [65,66], dominate over viral escape mechanisms in these patients.

Together our study raises the possibility that adaptation of Vpu to selective pressures differs between EC and CP HIV patients. Such adaptation could be paralleled by a moderate reduction of several biological activities of Vpu with the notable exception of antagonism of the particle release restriction mediated by CD317/tetherin, which is highly conserved. These results support that CD317/tetherin antagonism represents a cardinal Vpu activity in chronic HIV infection. The ability to adapt to selective pressures without disruption of this critical activity suggests that Vpu has evolved to spatially separate its functionally essential domains from those that provide plasticity for adaptation to the host environment.

## Supporting Information

S1 Fig Expression of patient-derived VpuIRESGFP proteins. Western Blot analysis of lysates of TZM-bl cells transfected with the indicated VpuIRESGFP expression constructs using antibodies against Vpu, GFP and transferrin receptor (TfR).
(TIF)

S2 Fig Subcellular localization of patient-derived Vpu. A3.01 cells were fixed on cover slips after 24 h post transfection with the indicated Vpu.GFP expression constructs. The plasma membrane was defined by staining with WGA-594. Cells in which degradation of Vpu.GFP was apparent by intense diffuse GFP fluorescence throughout the cells were excluded from analysis. Shown are representative confocal images (merge of red and green fluorescent channels). Scale bar = 10 μm.
(TIF)

S3 Fig CD4 and CD317 downregulation activity of VpuIRESGFP. Surface CD4 and CD317/tetherin levels were analyzed by flow cytometry on TZM-bl cells 48 h post transfection with the indicated VpuIRESGFP expression constructs. A, D: Flow cytometry plots of eGFP and NL4.3VpuGFP: CD4-APC, CD317/tetherin-APC (y-axis) vs. GFP (x-axis). Downregulation activity (MFI right gate/MFI left gate ratio) was normalized to NL4.3 Vpu that was arbitrarily set to 100%. B, E: CD4 /CD317/tetherin downregulation activity of patient derived Vpu alleles relative to NL4.3 Vpu. Shown are mean values of triplicate transfections with the indicated standard deviation. Results are representative of three independent experiments. C, F: Comparison of CD4/CD317/tetherin downregulation activity of EC and CP derived Vpu alleles. Statistical significance was assessed using the two-tailed Mann—Whitney U-Test (p = 0.009 (CD4) and 0.002 (CD317/tetherin)).
(TIF)
