# Supplementary figures and images for: Modest Attenuation of HIV-1 Vpu Alleles Derived from Elite Controller Plasma
Source: PLoS One. 2015 Mar 20;10(3):e0120434. doi: 10.1371/journal.pone.0120434 (PMC4368696; doi:10.1371/journal.pone.0120434)

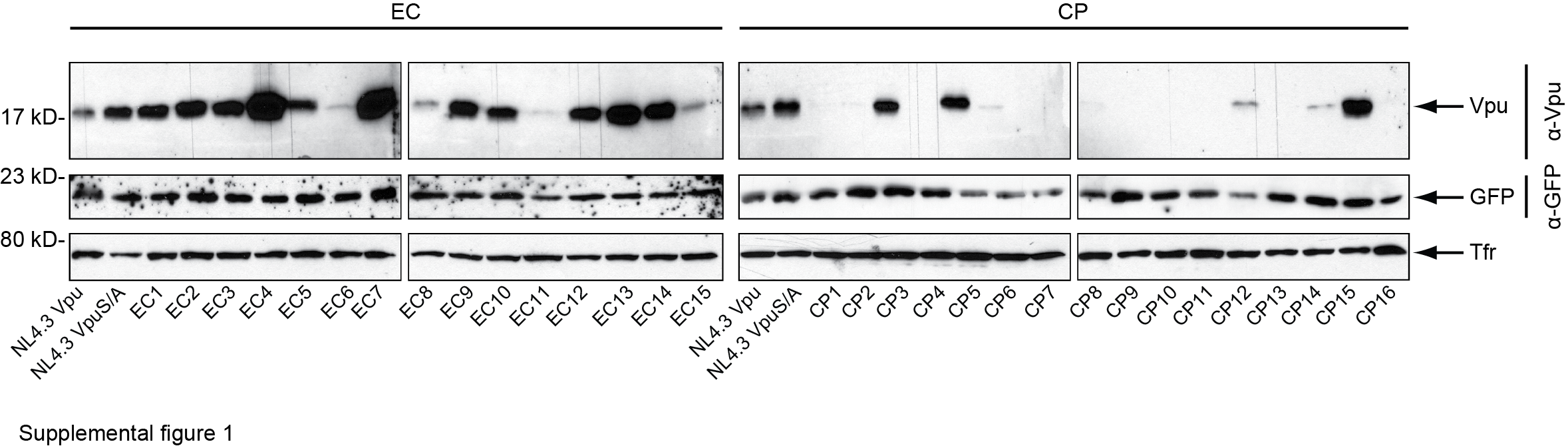

Supplement: S1 Fig — Western Blot analysis of lysates of TZM-bl cells transfected with the indicated VpuIRESGFP expression constructs using antibodies against Vpu, GFP and transferrin receptor (TfR). (TIF) [file pone.0120434.s001.tif]

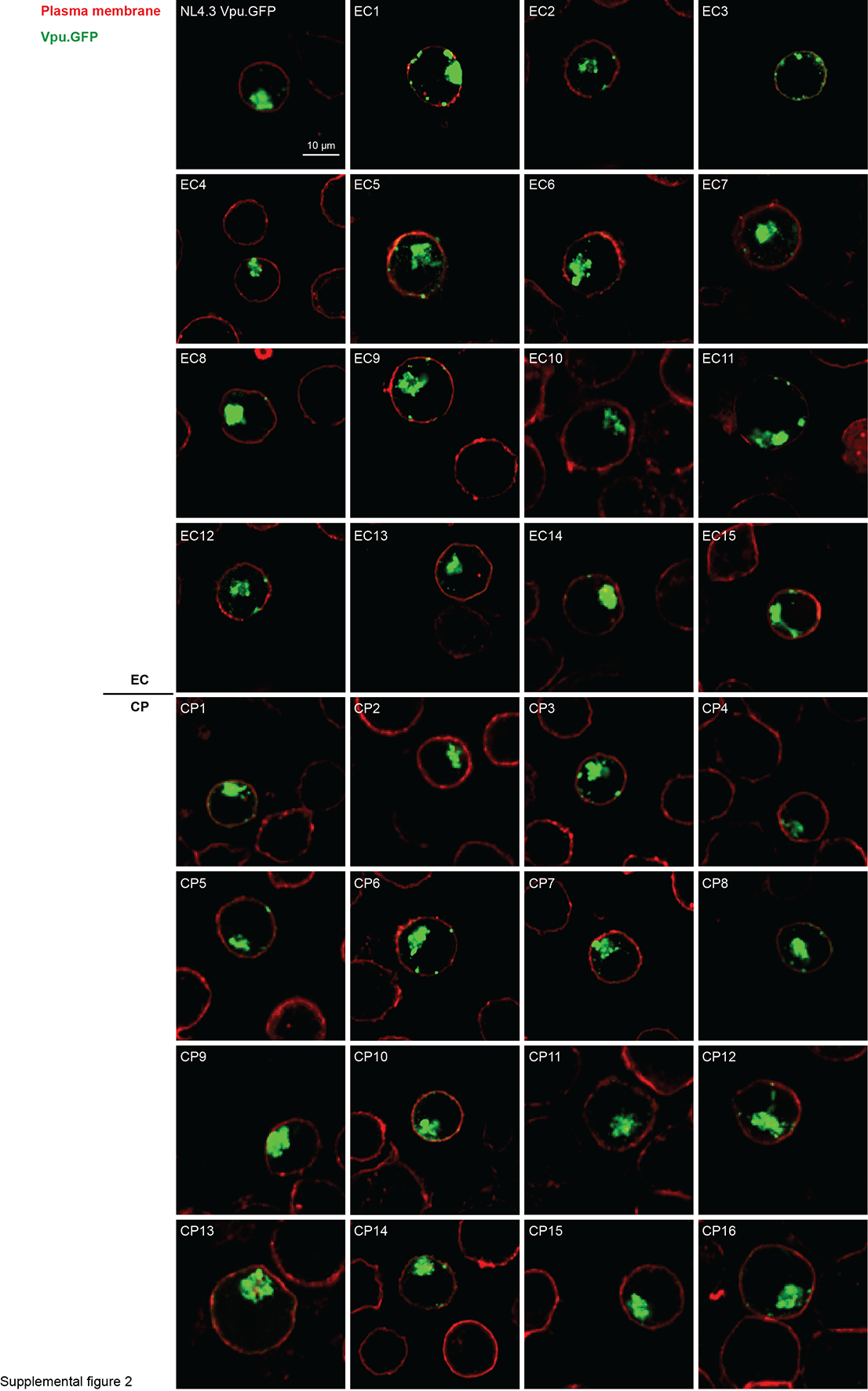

Supplement: S2 Fig — A3.01 cells were fixed on cover slips after 24 h post transfection with the indicated Vpu.GFP expression constructs. The plasma membrane was defined by staining with WGA-594. Cells in which degradation of Vpu.GFP was apparent by intense diffuse GFP fluorescence throughout the cells were excluded from analysis. Shown are representative confocal images (merge of red and green fluorescent channels). Scale bar = 10 μm. (TIF) [file pone.0120434.s002.tif]

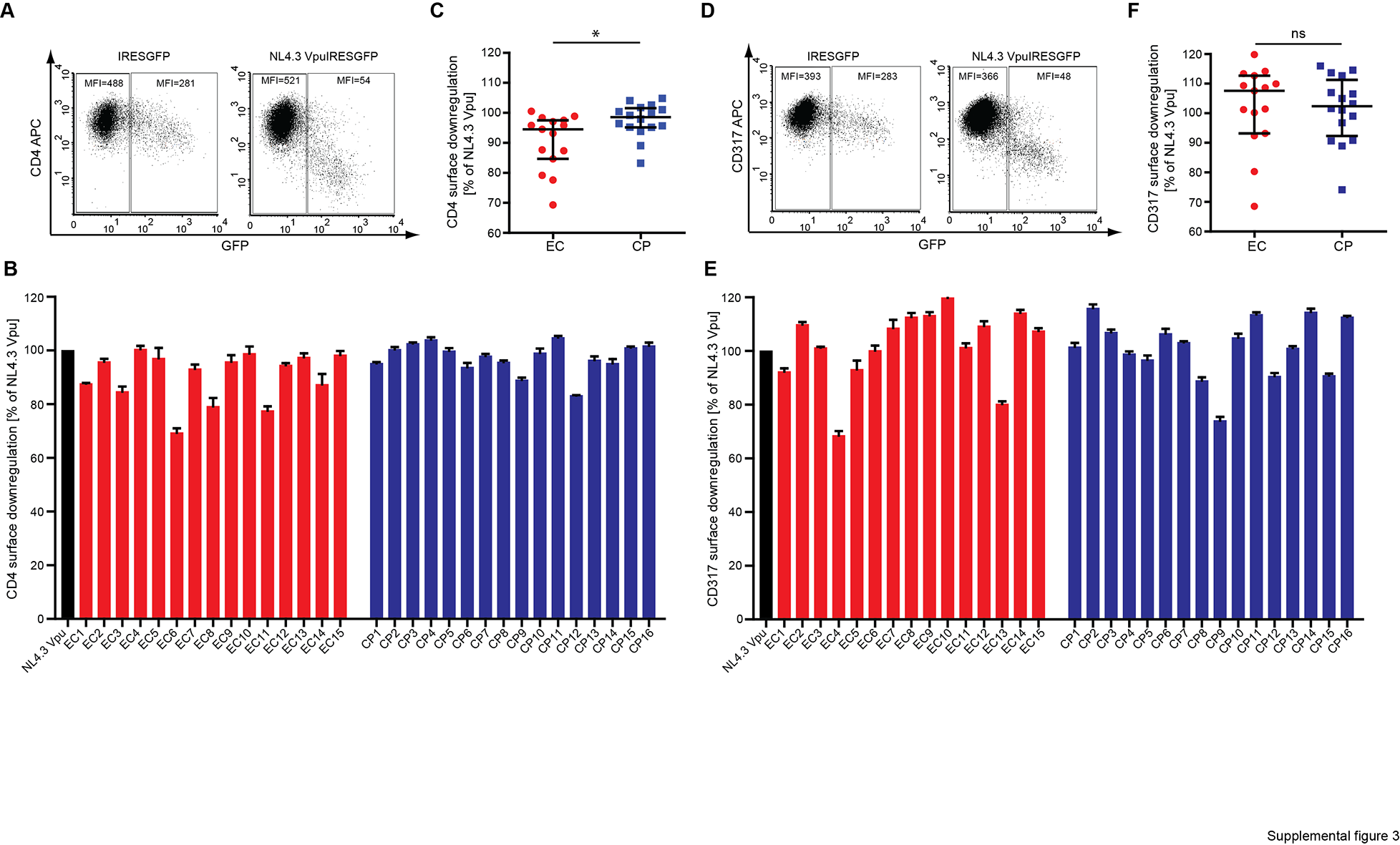

Supplement: S3 Fig — Surface CD4 and CD317/tetherin levels were analyzed by flow cytometry on TZM-bl cells 48 h post transfection with the indicated VpuIRESGFP expression constructs. A, D: Flow cytometry plots of eGFP and NL4.3VpuGFP: CD4-APC, CD317/tetherin-APC (y-axis) vs. GFP (x-axis). Downregulation activity (MFI right gate/MFI left gate ratio) was normalized to NL4.3 Vpu that was arbitrarily set to 100%. B, E: CD4 /CD317/tetherin downregulation activity of patient derived Vpu alleles relative to NL4.3 Vpu. Shown are mean values of triplicate transfections with the indicated standard deviation. Results are representative of three independent experiments. C, F: Comparison of CD4/CD317/tetherin downregulation activity of EC and CP derived Vpu alleles. Statistical significance was assessed using the two-tailed Mann—Whitney U-Test (p = 0.009 (CD4) and 0.002 (CD317/tetherin)). (TIF) [file pone.0120434.s003.tif]
